# Supplementary figures and images for: Inter-species variation in monovalent anion substrate selectivity and inhibitor sensitivity in the sodium iodide symporter (NIS)
Source: PLoS One. 2020 Feb 21;15(2):e0229085. doi: 10.1371/journal.pone.0229085 (PMC7034854; doi:10.1371/journal.pone.0229085)

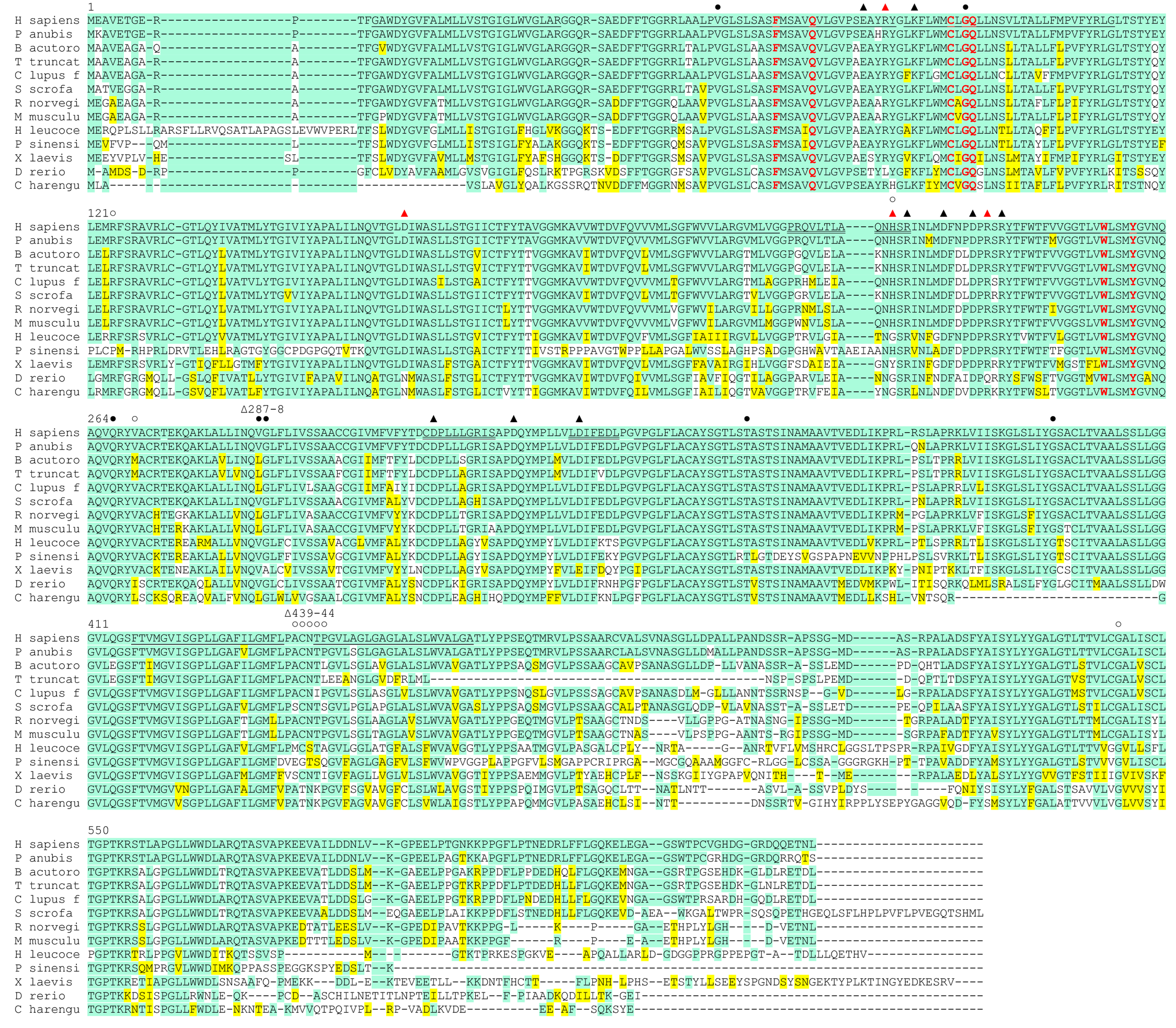

Supplement: S1 Fig — H. sapiens (human), P. anubis (olive baboon), B. acutorostrata scammoni (minke whale), T. truncatus (bottle-nosed dolphin), C. lupus familiaris (dog), S. scrofa (pig), R. norvegicus (rat), M. musculus (mouse), H. leucocephalus (bald eagle), P. sinensis (Chinese soft-shell turtle), X. laevis (African clawed frog), D. rerio (zebrafish), and C. harengus (Atlantic herring). Cyan highlighting indicates absolute conservation to human NIS. Yellow indicates similar residue to human NIS. Underline indicates putative transmembrane domain in human NIS, only TM1-12 are indicated. Closed circles indicate site of a mutation known to cause a transport defect in humans [22]. Open circles indicate site of a mutation known to cause membrane trafficking defect in humans [22]. Black triangles indicate a charged residue where mutation to alanine significantly reduces iodide uptake in human NIS [71]. Red triangles indicate a charged residue where mutation to alanine significantly reduces iodide uptake in human NIS and this residue is not charged in zebrafish NIS [71]. Bold red lettering indicates residue reported to be involved in stoichiometry control and translocation dynamics [52, 53]. Species are ordered in ascending evolutionary proximity to humans as determined by TimeTree (pig, mouse, rat, and dog diverged equidistantly) [73]. Numbering follows human NIS. (TIF) [file pone.0229085.s001.tif]

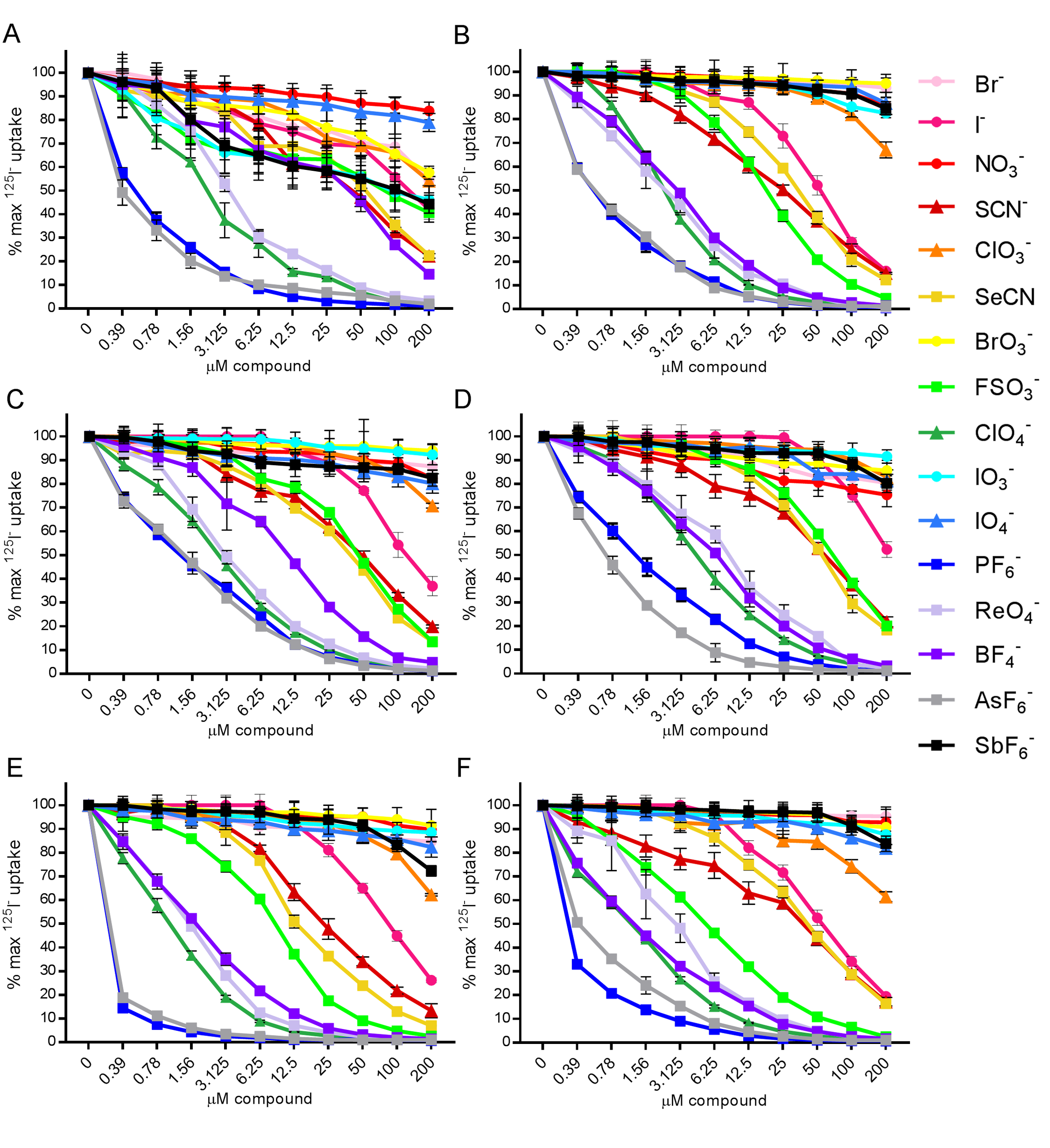

Supplement: S2 Fig — (A) HA-African clawed frog NIS, (B) HA-pig NIS, (C) HA-mouse NIS, (D) HA-rat NIS, (E) HA-dog NIS, or (F) HA-olive baboon NIS. Data shown as the percentage of maximum 125I- uptake activity (0 μM compound) maintained in the presence of increasing concentrations of substrate or inhibitor. Circular markers indicate naturally occurring anions. Triangular markers indicate anions which may occur naturally at low levels or are generated inside the organism. Square markers indicate anions not found naturally. Values are averages of duplicate assays with standard deviation. (TIF) [file pone.0229085.s002.tif]

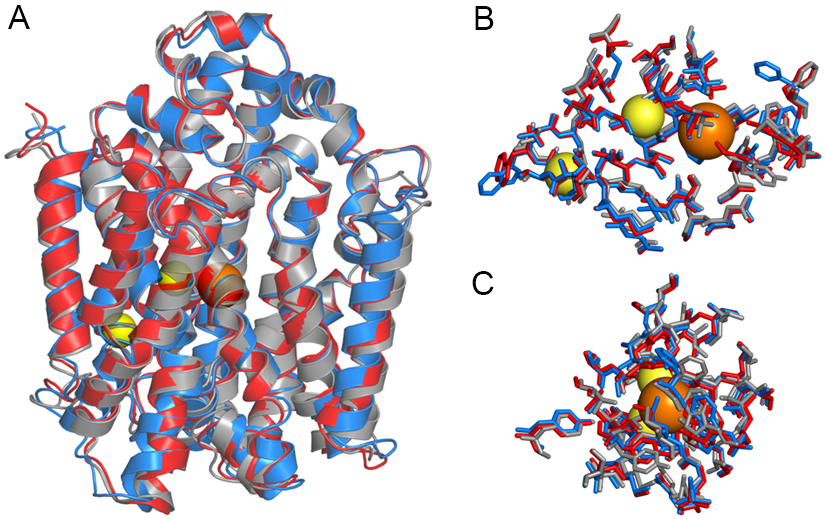

Supplement: S3 Fig — (A) Overlap of hNIS (red), wNIS (blue), and zNIS (grey). The Na+ and I- are presented as yellow and orange spheres, respectively. Color coding follows the colors used for Figs 3–5 and 7. (B-C) Two different projections of residues from the ion coordination spheres (within 5Å) identified from our MD simulations. The same projections were used in Fig 8D and 8E. (TIF) [file pone.0229085.s003.tif]
