# Supplementary material for: Inter-species variation in monovalent anion substrate selectivity and inhibitor sensitivity in the sodium iodide symporter (NIS)
Source: PLoS One. 2020 Feb 21;15(2):e0229085. doi: 10.1371/journal.pone.0229085 (PMC7034854; doi:10.1371/journal.pone.0229085)
Supplement: Attachment — Submitted filename: Response to Reviewers.docx [file pone.0229085.s004.docx]

Claudio M. Soares, Ph.D.

Academic Editor, *PLOS One*

January 27, 2020

Dear Dr. Soares,

I am writing to submit our edits and point by point response to the reviewer critique of our manuscript entitled “Inter-species variation in monovalent anion substrate selectivity and inhibitor sensitivity in the sodium iodide symporter (NIS)”, PONE-D-19-35941. We greatly appreciate the timely manner in which this review was conducted. We thank the editor-in-chief and Dr. Soares for their role in this process and assistance with the submission process. We also thank the reviewer for his/her detailed reading of our manuscript and his/her assessment of the strengths and weaknesses, as well as insightful suggestions.

We have edited the manuscript to adhere to *PLOS One*’s style and requirements. We have run our figures through the Preflight Analysis and Conversion Engine (PACE) and they meet *PLOS One*’s publication standards. We have also shortened the Materials and Methods, Results, and Discussion sections as much as possible, per Reviewer 1’s recommendation.

In regards to Reviewer 1’s specific comments, we have addressed the two major concerns with additional text in the manuscript.

1) Reviewer 1: *“[T]he relationship between substrate transport efficiency and iodide content in the environment may be less likely to reflect the comparative protein structure of NIS, and any evolutionary adaptations in terms of amino acid sequence, and more likely to reflect NIS expression levels and subcellular localisation. This point should be addressed.”*

To expand upon our addition to the discussion about this point, we believe our results are indicative of fundamental attributes of the NIS proteins examined in this study as we made every attempt to equalize cell surface expression both during the experiment via the creation of cell lines with near-identical expression and after the experiment by normalization of uptake values to expression levels as determined by flow cytometry of transfected and transduced cell lines. Any differences in substrate transport observed should be the result of inherent protein function rather than differences in protein maturation, cell surface expression, or subcellular localization.

2) Reviewer 1: *“Furthermore, human proteins known to bind NIS and alter its function may not interact with e.g. zebrafish NIS in the same way. Although it is not feasible to repeat the experiments in zebrafish and minke whale cell lines, the authors should comment.”*

We cannot deny the possibility that minke whale NIS and zebrafish NIS may have altered function due to being tested in a human cell environment. However, two points support our results and conclusions despite the potential human NIS bias: 1) As mentioned in the updated discussion, to date, there are no cellular factors known to bind or interact with NIS which affect the ion transport function. Two papers have reported NIS interacts with leukemia-associated RhoA guanine exchange factor (LARG) (DOI:10.1158/0008-5472.CAN-12-0516 and DOI: 10.1158/0008-5472.CAN-18-1954). However, this interaction was not shown to have any affect upon NIS transport activity and is considered a nonpump function of NIS. 2) Figure 2E and 2F indicates that human-, minke whale-, and zebrafish NIS are capable of transporting iodide to the same degree, suggesting there is no restriction of minke whale- or zebrafish NIS function in human cells. We also acknowledge that this manuscript does not provide experimental evidence of a mechanism for the observed differences, but we are confident that the advances we offer will be the source of much discussion and future work in the NIS field.

We are hopeful that this manuscript will be accepted. As such, we will upload our data into a public repository concurrently with the resubmission review and share the accession numbers or DOIs immediately upon acceptance.

We have read the journal's policy and the authors of this manuscript have the following competing interests: S.J.R. is a cofounder and holds equity in Imanis Life Sciences, LLC. S.C.C., H.R.Z., and S.Y.N. declare no competing interests. This does not alter our adherence to PLOS ONE policies on sharing data and materials.

We look forward to hearing from you.

Sincerely,

Stephen J. Russell, M.D., Ph.D.

Corresponding Author

Professor of Medicine, Department of Molecular Medicine, Mayo Clinic

200 1^st^ St SW Rochester, Minnesota, 55905 USA

sjr@mayo.edu; Tel: 1-507-774-5373
